# Antipsychotics in off-label use: prescription practices, benefits and risks Results from APSY Oulu study

**DOI:** 10.1192/j.eurpsy.2023.648

**Published:** 2023-07-19

**Authors:** E. Jääskeläinen

**Affiliations:** Research Unit of Population Health, University of Oulu, University of Oulu, Finland

## Abstract

**Introduction:**

Use of antipsychotics (APs) has increased worldwide during last decades. Main reason for this is off-label use, and especially the use of quetiapine for insomnia and anxiety. There is an insufficient amount of knowledge about the prescription habits and the effects of APs in off-label use. For example, APs have shown efficacy in the treatment of anxiety, but long term follow-ups are rare. Only two trials on effects of quetiapine in primary insomnia exist, without much evidence. Some small studies have indicated that even relatively small doses of APs may cause side-effects, such as increased weight and metabolic changes.

**Objectives:**

The aim of the APSY Oulu project is to analyse the prescriptions and use of APs off-label, and benefits and risks of APs in off-label use.

**Methods:**

In 2019 a questionnaire study for doctors in different health care organisations in Finland was preformed. The purpose of the questionnaire was to find out the physicians’ prescription habits, thoughts and experiences concerning APs, especially in off-label use. In ongoing clinical study we will investigate whether the use of most frequently used off-label AP quetiapine will associate to changes in participants’ overall health, mental symptoms or cognitive functions during 6-12 months follow-up. In addition, in general population sample, we will compare characteristics and clinical outcomes of 137 persons being prescribed APs off-label and comparison groups.

**Results:**

Based on questionnaire for Finnish physicians (n=216), APs off-label prescriptions are mostly for insomnia and anxiety, most common drug being quetiapine. APs are being prescribed off-label by GPs, occupational health doctors and psychiatrists. The monitoring of metabolic values was not very common: 44% of the psychiatrists and 18% of other physicians reported to follow-up metabolic values of the patients (Pentinlehto J et al. Psychiatria Fennica 2021;52:22-). We have collected pilot sample of 10 individuals starting quetiapine for insomnia. They all had severe or very severe symptoms of insomnia, and used very small dose of quetiapine (mostly 12,5-25mg). Four of them used quetiapine still at the 12 months follow-up, and their insomnia symptoms decreased during the follow-up. Side effects were common (e.g. increase heart rate, drowsiness) and caused discontinuation of quetiapine for some persons. In the general population based Northern Finland Birth Cohort 1966 individuals who had been prescribed APs off-label had poorer health, lower socioeconomic status, consumed more alcohol and smoked more often compared with individuals with non-psychotic mental disorders without APs off-label (Pirhonen E et al. Acta Psychiatr Scand. 2022;146:227-).

**Conclusions:**

Off-label prescriptions and use of APs is common. Further studies on APs off- label use and its safety are needed. There is a need for guideline on monitoring the patients during APs in off-label use.

**Disclosure of Interest:**

None Declared

